# The acetotrophic pathway dominates methane production in Zoige alpine wetland coexisting with hydrogenotrophic pathway

**DOI:** 10.1038/s41598-019-45590-5

**Published:** 2019-06-24

**Authors:** Yanfen Zhang, Anzhou Ma, Guoqiang Zhuang, Xuliang Zhuang

**Affiliations:** 1 Key Laboratory of Environmental Biotechnology, Research Center for Eco-Environmental Sciences, Chinese Academy of Sciences, Beijing, 100085 China; 2 University of Chinese Academy of Sciences, Beijing, 100049 China

**Keywords:** Microbial communities, Biogeochemistry

## Abstract

As a typical alpine wetland on the Tibetan Plateau, the Zoige wetland processes a large carbon stock and is a hotspot of methane emission. To date, many studies have investigated the methane flux in this wetland; however, the research on the source of methane in the soils of Zoige wetland is not clear enough. In this study, we determined the dynamic characteristics of the stable carbon isotopes during the methanogenesis of Zoige wetland soil and the corresponding microbial changes. The results showed that the δ^13^CH_4_ varied between −19.86‰ and −28.32‰ and the α_*C*_ ranged from 1.0029 to 1.0104 in the methanogenesis process, which suggests the dominance of acetotrophic methanogenesis. And among the increased methanogens, acetotrophic methanogens multiplied more obviously than hydrogenotrophic menthanogens. In addition, the results of structural equation models showed that the variations in stable carbon isotopes during the process were mainly affected by acetotrophic methanogens. Although the acetotrophic pathway was dominate, the varied isotope characteristics, increased methanogens and ratio of carbon dioxide to methane all showed that hydrogenotrophic and acetotrophic methanogenesis coexisted in the Zoige wetland. Overall, our study provided a detailed and definitive information to the source of methane in the soil of the Zoige wetland and laid a foundation of mechanism to the research of greenhouse gas in this alpine wetland.

## Introduction

As an important greenhouse gas, methane has attracted a great deal of attention under the background of global warming. According to studies of methane sources, biogenic methane contributes almost 69% of global methane emissions^1^. In biological processes, methane is the end product of organic matter degradation under anaerobic conditions and is produced by a group of specialized microorganisms known as methanogens. The methanogens have a unique enzyme named methyl-coenzyme M reductase (Mcr) that makes them specialized for produce methane. Few substrates are directly used for methanogenesis; namely, acetate, carbon dioxide, and some methylated compounds. Moreover, methane from different substrates has different characteristics and is generated by different methanogenic microorganisms.

Based on differences among substrates, the methanogenic process can be divided into acetotrophic pathway, hydrogenotrophic pathway and methylotrophic pathway^2^. The acetotrophic and hydrogenotrophic pathways have been reported to be the main methanogenic pathway in most environments^3^, while the methylotrophic pathway mainly exists in some specific ecological environments, such as salt lakes^4^ and coal seams^5^. Usually, the methane-producing pathways in the environment are determined by sequencing the corresponding methanogenic microorganisms^6^. However, the presence of methanogens cannot ensure the activity of these methanogens in the environment. Since the ratios of contributions from different pathways differ, the methane produced usually exhibits different carbon isotopic fractionation characteristics^7,8^. Therefore, determining the composition of methane isotopes can more accurately reflect the methanogenic pathways in the environment and provide more complete evidence together with the results of microbiological analysis^7^. Considering the effects of substrate type, substrate concentration, and active compositions of methanogens on the isotopic fractionation and the heterogeneity of the environment, the fractionation factors for each methanogenic environment should be determined to establish their methanogenic pathways^8^.

Wetlands have been shown to be the largest source of methane, contributing 20–39% to the global emissions^9,10^. Due to the in-depth understanding of the relationship between methane and warming^11,12^, wetlands in high-latitude and high-altitude regions have drawn increasing attention because of their sensitivity to climate change^13^ and large carbon stock^14^. The Zoige wetland, located on the northeast region of the Tibetan Plateau, is a typical high-latitude wetland. The carbon reserves of this wetland account for 6.2% of the total carbon storage in China^15^ and this wetland has also been reported to be a hotspot of methane emissions in many studies^16–19^. To investigate the methanogenic pathways in this wetland, the addition of exogenous substrates combined with microbial analysis has been used in most studies^20–22^. However, those cannot reveal the methanogenic activity of the soil itself in the Zoige wetland. Therefore, in this study, we determined the methanogenic pathways in the Zoige wetland through analysis of both stable carbon isotopes and microorganisms in the methanogenic process without any exogenous addition.

The objective of this study is to find out the methane sources in the soils from the Zoige alpine wetland through monitoring the dynamics of carbon isotopic compositions and analyzing the corresponding microbial changes during the methanogenic process. To accomplish this, anaerobic microcosm experiments were designed to incubate soils from the Zoige alpine wetland at *in situ* temperature without any exogenous additions. The relevant metabolites and stable carbon isotope compositions of methane and carbon dioxide were then measured during the methanogenic process. In addition, the microbial variance was studied based on quantification of Mcr α-subunit genes (*mcrA*) and high-throughput sequencing of archaeal 16S rRNA genes.

## Results

### Methane production potential

**Figure 1:**
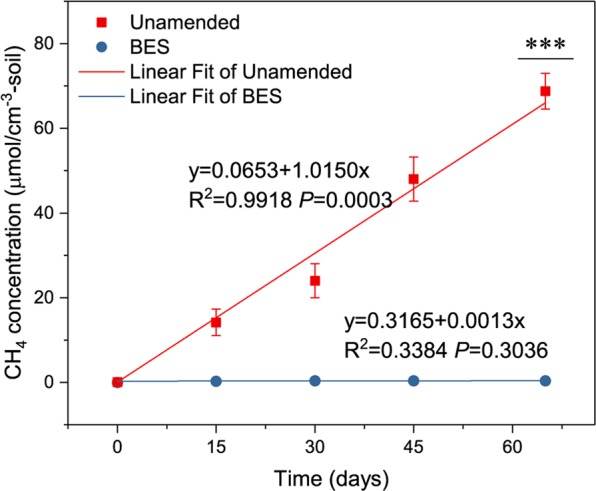
Methane production potential of soils from the Zoige alpine wetland. The values are shown as the means ± s.e.m., n = 3.

Under anaerobic conditions, without any additions, soils from the Zoige alpine wetland showed clear methane production potential (Fig. 1). The methane concentration increased gradually during the entire process and showed a significant correlation with the incubation time (*P* ≤ 0.001). While, the slope of the BES group after linear fitting was not significantly different from 0 (*P* ≥ 0.05). At the end of the incubation period (65 days), the methane concentration was significantly higher than that of the BES-added control group (*P* ≤ 0.001). Moreover, the slope of methane production fitting curve in the unamended group was significantly different (*P* ≤ 0.001) from that of BES group after the univariate analysis of variance with a methane production rate of 1.015 μmol·day^−1^·cm^−3^ soil.

### Stable carbon isotope signatures during the methane production process

**Figure 2:**
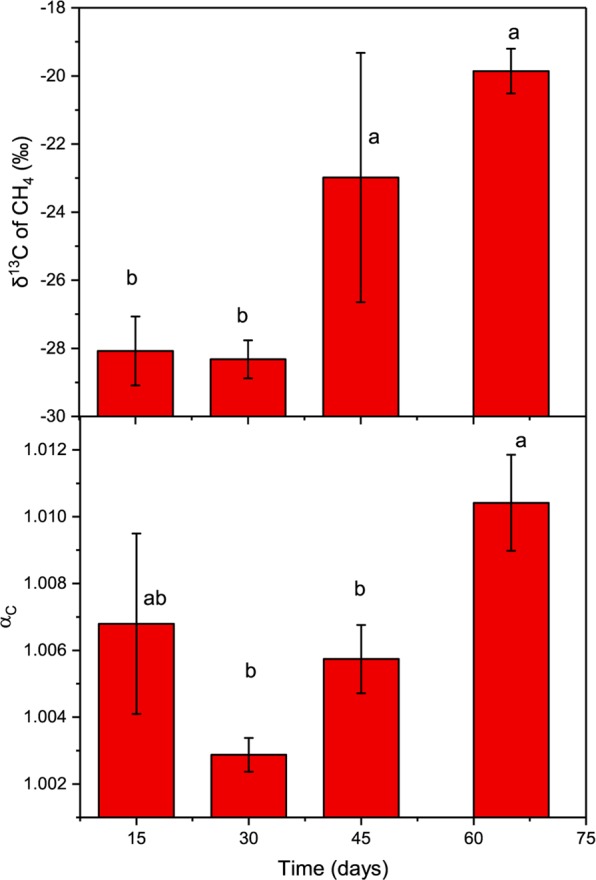
The δ^13^CH_4_ and apparent isotopic fractionation factor (α_*C*_) during the methane production process in the unamended group. The values are shown as the mean ± s.e.m., n = 3.

The stable carbon isotope signatures of CH_4_ and CO_2_ were measured during the methane production process (except time 0). The δ^13^CH_4_ value ranged between −19.86‰ and −28.32‰, with values at day 15 and day 30 significantly lower than that at day 45 and day 65 (Fig. 2). Based on the measured δ^13^CH_4_ and δ^13^CO_2_, the apparent carbon isotope fractionation (α_*C*_) for this methanogenic process was calculated according to formula: α_*C*_ = (δ^13^CO_2_ + 1000)/(δ^13^CH_4_ + 1000)^23^. The α_*C*_ was lowest at day 30 with the value of 1.0029. And the highest value was 1.0104 at day 65, which was significantly higher than that at day 30 and day 45.

### Variations in acetate concentration variation

**Figure 3:**
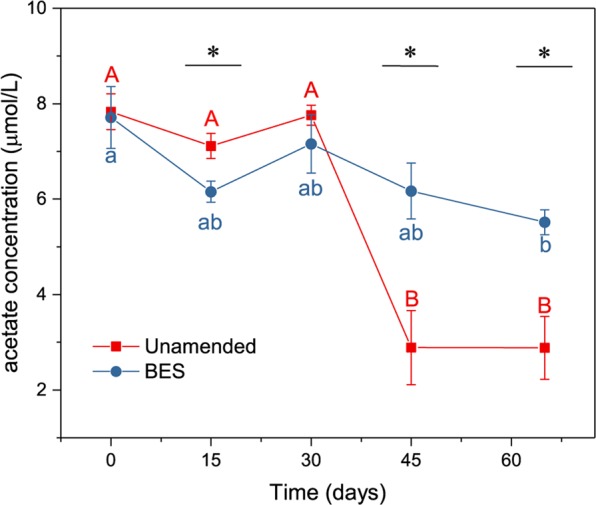
The concentration of acetate during the methane production process. The values are shown as the mean ± s.e.m., n = 3.

During the methane production process in the unamended group, the acetate concentration was about 2.88–7.83 µmol/L (Fig. 3) and the acetate concentration at days 45 and 65 significantly decreased (0.05 level) with the initial acetate concentration. After the methane production process was inhibited with BES-added, only the concentraion at days 65 showed significant decrease with the initial acetate concentration after one-way analysis of variance (0.05 level). After T test on the data at each time point, the acetate concentration at days 15, 45 and 65 all showed significant difference between the unamended and BES group (Fig. 3).

### Methanogens during the methanogenic process

**Figure 4:**
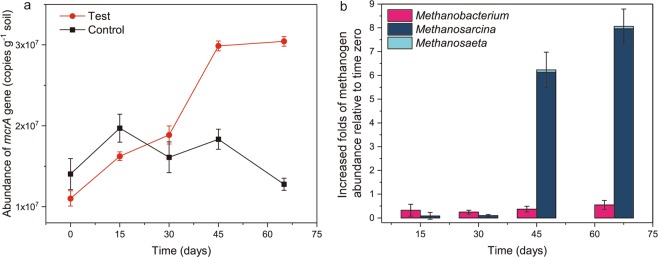
Changes in methanogens. (**a**) The abundance of *mcrA* genes; (**b**) the increased folds of methanogen abundance relative to time zero in the unamended group. The values are shown as the mean ± s.e.m., n = 3.

**Figure 5:**
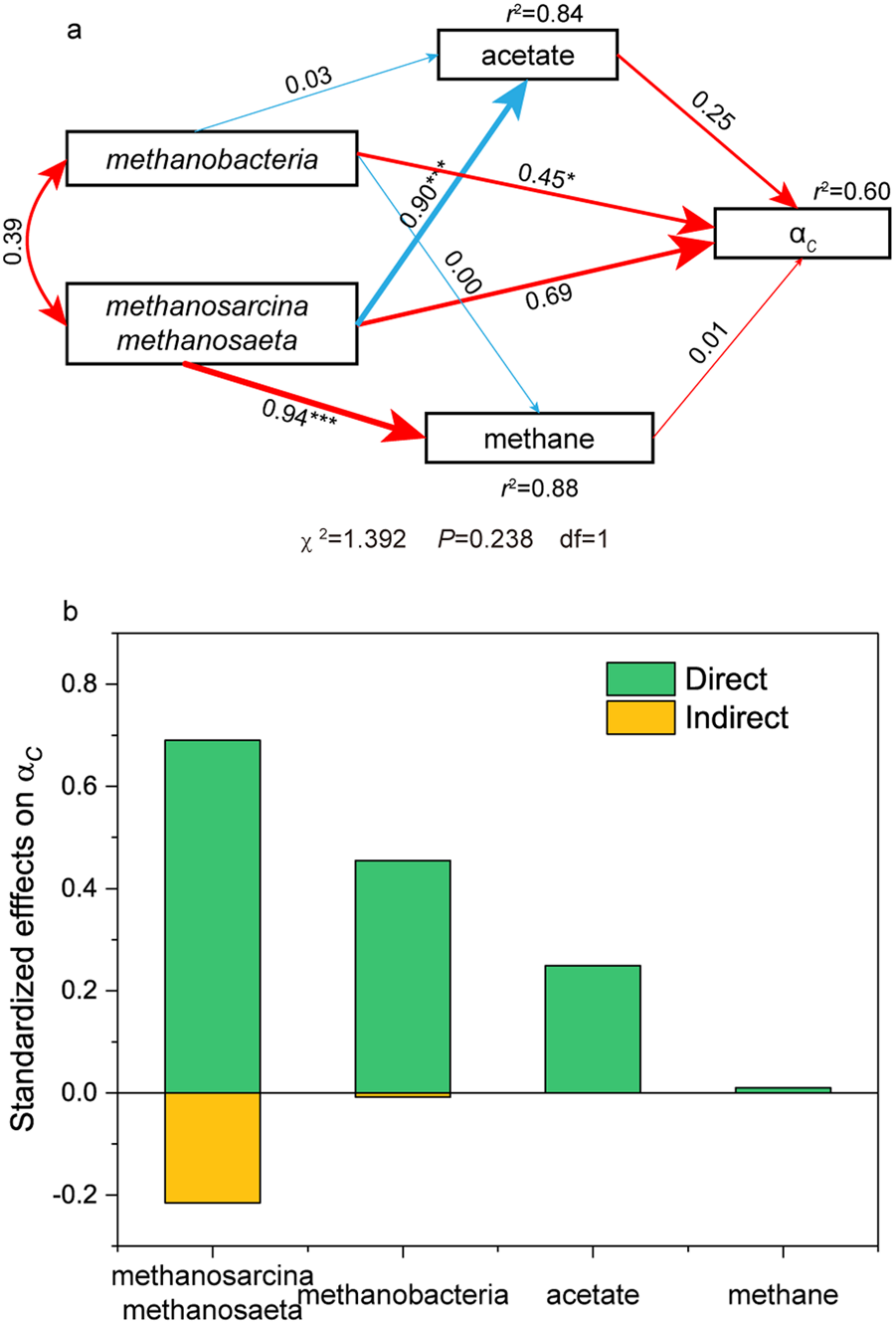
Structural equation model analysis (SEM) examining the effects of methanogens on acetate, methane and α_*C*_ in the methane production process of unamended group (**a**) and the standardized effects of *methanobacteria*, *methanosarcina* and *methanosaeta*, acetate and methane on α_*C*_ (**b**). Numbers adjacent to arrows indicate the effect-size of the relationship. The width of arrows is proportional to the strength of the relationship. The red arrow means positive relationship and the blue arrow means negative relationship. The r^2^ denotes the proportion of the response variables explained by relationships with other variables. n = 12. *p ≤ 0.05, **p ≤ 0.01, ***p ≤ 0.001.

Quantitative PCR (qPCR) analysis of *mcrA* gene showed that (Fig. 4a) the methanogens increased with time during the methane production process, while the methanogens in the inhibited treatment fluctuated at low levels. High-throughput sequencing revealed that the methanogens throughout the process were mainly *Methanobacterium* sp., *Methanosarcina* sp., and *Methanosaeta* sp., with an abundance ratio of 97% to all the methanogens. When compared to time 0, the relative abundance of *Methanobacterium* sp. increased slowly during the process to an abundance of 0.55 times greater than that at the beginning of the process. Moreover, the relative abundance of *Methanosarcina* sp. and *Methanosaeta* sp. both increased quickly after 30 days during the methane production process (Fig. 5b).

### The effects of different methanogens on δ^13^CH_4_ and α_*C*_

To analyze the effects of increased methanogens on the varied stable carbon isotope compositions during the methane production process in unamended group, a structural equation model (SEM) was built among the relative increased abundance of *Methanobacterium* sp., acetotrophic methanogens, acetate, methane and α_*C*_. The final model had a good fit to the datasets according to the Chi-squared test (Fig. 5a), which showed a ratio of χ^2^/*df* ≤2 and *P* > 0.05^24^. In the model, the *Methanosarcina* sp. and *Methanosaeta* sp. showed a significant positive effect on methane and significant negative effect on acetate. The *Methanobacterium* sp. had little effects both on methane and acetate. While, the direct effect of *Methanobacterium* sp. on α_*C*_ was significant, although it was lower than that of *Methanosarcina* sp. and *Methanosaeta* sp. on α_*C*_. After all the effects on α_*C*_ were taken into account (Fig. 5b), the acetotrophic methanogens were found to show the most important effects on α_*C*_, and the *Methanobacterium* sp. followed.

## Discussion

The Zoige wetland, located on the northeast portion of the Tibetan Plateau, has been reported to possess a huge carbon stock^15^ and to be a hotspot of methane emissions^16^. However, few studies have investigated the dynamic features of the methanogenesis process of the soil and revealed where the methane come from without any exogenous additions in the Zoige wetland.

In the current study, soils from the Zoige alpine wetland showed clear methane production potentials under *in situ* temperature conditions and without any additions as precursors. The typical methane production potential for wetlands was reported to vary from 10^−2^ to 10^1^ µmol·s^−1^·m^−3^ ^25^, and the rate in the current study was converted to11.57 µmol·s^−1^·m^−3^, which was typical but relatively high. This may have been because of the abundant organic materials in the peat soil of the Zoige wetland^26^, similar to the high methane production potentials reported in other peatlands^27,28^.

After an analysis of the stable carbon isotope compositions, we found that the methane produced in this methanogenesis process was rich in ^13^C, as the values of δ^13^CH_4_ in the current study were between −28.32‰ and −19.86‰ and the reported values of δ^13^CH_4_ from the biogenic process always ranged from −110 to −20‰^7,23,29^. Among all of the methanogenic pathways, acetotrophic and hydrogenotrophic methanogenesis were reported to be the dominant pathways in most environments, including wetlands. Moreover, methane produced by different pathways was found to have different isotope characteristics^7^. The value of δ^13^CH_4_ from acetate is usually higher than that from carbon dioxide in hydrogentrophic pathway, and assumed to vary from −60‰ to −20‰^7,29^. This suggests that the methane in the current study was mainly from the acetotrophic pathway, which has also been found to exist in the Zoige wetland in two studies of the warming effects on methane production^13,30^.

In the acetotrophic pathway, the carbon from acetate also forms CO_2_ besides CH_4_^31^. In our test for methane production, remarkable CO_2_ was produced accompanied with production of methane (Fig. S1a). And, it is worth noting that the CO_2_ production was also inhibited in the control group which was treated by specific inhibitor for methane production. Moreover, the produced CO_2_ concentration was significantly correlated with the CH_4_ concentration (Fig. S1b). These suggests that the source of carbon dioxide is closely related to the production of methane. In other words, the large amount CO_2_ may be the product of methane oxidation or the by-product of methane production. While, the addition of extra methane from the beginning of the incubation did not significantly change the amount of carbon dioxide produced (Fig. S1c). This means the produced large amount of CO_2_ was not from methane oxidation. In other words, the produced CO_2_ in our test was mainly the by-product of methane production, which is consistent with our speculation that the produced methane mainly comes from the acetotrophic pathway.

In addition to the carbon stable isotope characteristics of methane, the carbon stable isotope characteristics of carbon dioxide also has been considered to distinguish the pathway of methane production^23^. It is used as a complex index, α_*C*_, which combined the value of δ^13^CH_4_ and δ^13^CO_2_. For α_*C*_, a value higher than 1.065 is usually characteristic of environments dominated by hydrogenotrophic methanogenesis, while a value lower than 1.055 is characteristic of an environment dominated by acetotrophic methanogenesis^23,32^. Therefore, from the results in our test (Fig. 2), we can inferred again that acetotrophic methanogenesis is the main pathway during the methane production process.

Although dominant in soils from the Zoige wetland, acetotrophic methanogenesis may not be the only process leading to methane production in this system. This is because, although maintained in the levels of acetotrophic pathway, the values of δ^13^CH_4_ and α_*C*_ fluctuated during the process. The increased δ^13^CH_4_ after 30 days indicates an increased ratio of methane produced from acetate^33^. And the determination of acetate in this process showed that acetate significantly decreased at the same time, which is consistent with the variance of δ^13^CH_4_ and confirmed the high activity of acetotrophic pathway. Along with the increase of δ^13^CH_4_, the α_*C*_ should have decreased according to the formula. However, the α_*C*_ in the current study increased after 45 days. This was due to an increase of δ^13^CO_2_ during this process, which may be the result of methanogenic consumption of CO_2_ discriminating against ^13^C in the hydrogenotrophic pathway^34^. This inference is not only based on the common reports that acetotrophic and hydrogenotrophic methanogenesis generally coexist in most environments, but also based on the ratio of carbon dioxide to methane in this study (Fig. S2b). The theoretical ratio of carbon dioxide to methane in acetotrophic pathway should be 1:1, but 0.52:1 in our study. Assumed the low value were due to the consumption of carbon dioxide by hydrogenotrophic methanogens, then the measured amount of carbon dioxide should then be the difference between the acetotrophic pathway and the hydrogenotrophic pathway. Take the total produced methane as 1, and the ratio of acetotrophic pathway as x and hydrogenotrophic pathway as y, then x minus y should be equal to 0.52 and x plus y should be less than 1. Then, after solving the equation, the ratio of acetotrophic pathway during the methane production process should be less than 76%. This ratio is reasonable to the theoretical ratio 67%^8,32^ and suggests the dominating of acetotrophic pathway in the soils with two pathway coexisted.

After an analysis of the microorganisms, hydrogenotrophic and acetotrophic methanogens were also found to coexist during the process. Along with the production of methane, the abundance of methanogens has been increasing throughout the entire methanogenic process (Fig. 4a). During this process, there was rapid growth between days 30 and 45, which is consistent with the increased methane production rate. Based on the composition of methanogens, the microorganisms responsible for this rapid growth were mainly *Methanosarcina* sp. and *Methanosaeta* sp. (Fig. 5b), which both showed a high rate of multiplication during this period. Moreover, these organisms are the two main genera that can grow and produce methane with acetate as the substrate^35^. Hence, the increased abundance of these organisms may led to a decrease in acetate concentration at the corresponding time points, which is consistent with the result of acetate concentration in this study. Another dominant methanogen, *Methanobacterium*, is a typical hydrogenotrophic genus that produces methane^36^. During the methane production process, the relative abundance of *Methanobacterium* sp. also increased, but at a slower rate than *Methanosarcina* sp. and *Methanosaeta* sp. This provides the microbial evidence for our previous interpretation of the increased δ^13^CH_4_ after days 30. Moreover, the increasing abundance of both acetotrophic and hydrogenotrophic methanogens again confirmed the coexistence of the acetotrophic and hydrogenotrophic pathway in the Zoige wetland. Although they coexisted, the acetotrophic methanogens had significant greater effects on methane than hydrogenotrophic methanogen (Fig. 5). This suggests that the methane produced in this process is mainly from the acetotrophic pathway via active acetotrophic methanogens.

In summary, this study revealed the dynamic characteristics of stable carbon isotope fractionation and the active methanogens in the methane process and demonstrated that acetotrophic and hydrogenotrophic methanogenesis coexist in the Zoige wetland with the acetotrophic pathway dominating. Although our study on the methane production pathway of the soils in the Zoige alpine wetland were conducted without any exogenous additions and incubated at *in situ* temperature, there was still a certain distance from the ecological reality at the Zoige wetland. Therefore, more in-depth and detailed studies are needed to reveal the complex dynamics and real ecological mechanism of methane production process in the Zoige alpine wetland.

## Methods

### Soil samples

The soil samples used for this study were collected from the Zoige National Wetland Reserve in 2015. To represent the entire wetland, five sites were set and samples for every site were collected in triplicate. After collection, all of the samples were transported to the laboratory on ice in sterile bags, where they were then stored at −20°C. The pH and temperature of each site were measured using a portable multi-parameter water analyzer (WTW, Multi340i, German) on site and the details are reported in Supplementary Table S1.

### Incubation experiments

Soil samples were mixed and diluted with 1:1 sterile Ar-flushed water in an anaerobic box. The resulting slurry was then homogenized and roots in it were removed. The slurry was subsequently split into 45 120-mL serum vials with 10 mL in each vial that were then sealed with rubber stoppers. Vials were taken out from the anaerobic box and evacuated with a vacuum pump for 5 min, back-flushed with high purity Ar five times, and subsequently filled with Ar to ambient pressure after the fifth cycle. Vials were then stored in the dark at 14 °C for 20 days to allow any trace amounts of O_2_ that might have remained to be consumed. Following the pre-incubation period, vials were evacuated and flushed again with Ar, after which they were divided into four groups with different treatments: (i) Unamended; (ii) BES (Sodium 2-Bromoethanesulfonate, specific inhibitor for inhibiting methane production); (iii) CH_4_. The group (i) was set for the determination of methanogenic capacity with group (ii) as control. The group (iii) was set to test whether the methane was consumed after it was produced in the system. The final concentration of BES was 50 mM and the Ar-purged stock solution was injected via disposable sterile syringes that were fitted with sealed stopcocks. Next, the 5 mL of headspace gas in each vial was removed and replaced with an equal volume of CH_4_ or Ar via syringes that were fitted with sealed stopcocks and flushed 4 times with ultrahigh purity Ar. The experiment employed destructive sampling, with three vials set for each treatment and measurement time. The headspace gas was sampled via syringes and stored in vacuum Labco Extainers until analysis, while the slurries were split into centrifuge tubes that were stored in an anaerobic box until further analysis.

### Analytical measurements

Headspace CH_4_ and CO_2_ concentrations were determined using a gas chromatograph mass spectrometer (Shimadzu, GCMS QP 2010 Ultra, Japan) equipped with a GS-CARBONPLOT column (Agilent, USA) in scanning mode. Helium was applied as the carrier gas with a pressure of 38 KPa and a split ratio of 29. The inlet temperature was 100 °C, the column temperature was set at 50 °C and the ion source temperature at 200 °C. The δ^13^C of methane and carbon dioxide were determined using a Delta v Advantage gas chromatograph combustion isotope ratio mass spectrometer system (GCC-IRMS) (Thermo Scientific, USA)^34^. The analytical procedures were the same as those for GC-MS. The apparent isotopic fractionation factor was determined by α_*C*_ = (δ^13^CO_2_ + 10^3^)/(δ^13^CH_4_ + 10^3^)^7^, while the concentrations of acetate in the slurries of the incubations were analyzed as previously described^13^.

### DNA extraction and real-time quantification PCR

DNA was extracted as previously described^37^. The functional gene in methanogenic pathway, *mcrA*, was quantified using the primer combination MLf/MLr^38^. An amplified *mcrA* sequence that had been sequenced and confirmed (NCBI accession number: MH102312) was used to construct the plasmid to generate a standard curve using the Lethal Based Fast Cloning Kit (Tiangen Biotech (Beijing) Co., Ltd.). The plasmid concentration was determined by spectrophotometric measurement with a Smartspec 3000 (BioRad) at 260 nm and then calculated according to the molecular mass. The standard curve was constructed from 1.931 × 10^9^ to 1.931 × 10^2^ copies/µL with 10-fold dilution steps. Samples in 5-fold dilutions were quantified in triplicate and repeated in at least two independent qPCR runs. A 25 µL reaction system was constructed using SYBR^®^ Premix Ex Taq™ (Tli RNaseH Plus) (TaKaRa, Dalian, China) and analyzed in BioRad CFX96 Connect Real-Time System (BioRad, Hercules, CA, USA). The thermal progress was as follows: 95 °C for 30 s, followed by 40 cycles of 95 °C for 5 s and 59 °C for 30 s. The melting curve was from 65 °C to 95 °C with an increment of 0.5 °C for 5 s.

### High-throughput Illumina sequencing

The archaea 16S rRNA genes were amplified using primer Arch519F/Arch915R with barcodes to identify species composition. All of the PCR reactions were conducting using Phusion^®^ High-Fidelity PCR Master Mix (New England Biolabs) and the products were purified with a Qiagen Gel Extraction Kit (Qiagen, Hilden, Germany). Sequencing libraries were generated using a TruSeq^®^ DNA PCR-Free Sample Preparation Kit (Illumina, San Diego, CA, USA) following the manufacturer’s recommendations and index codes were added. The library quality was assessed using a Qubit^®^ 2.0 Fluorometer (Thermo Scientific, Waltham, MA, USA) and the Agilent Bioanalyzer 2100 system. Finally, the library was sequenced on an Illumina HiSeq2500 platform and 250 bp paired-end reads were generated.

### Data analysis

The produced paired-end reads were assigned to samples based on their unique barcode, after which they were truncated by cutting off the barcode and primer sequence. The paired-end reads were then merged using FLASH (V1.2.7, http://ccb.jhu.edu/software/FLASH/) and the splicing sequences were considered as raw tags. After removing the chimera sequences with the UCHIME (http://www.drive5.com/usearch/manual/uchime_algo.html) algorithm, the effective tags were obtained. The effective tags were submitted to the NCBI Sequence Read Archive database under accession number SRP142729. Sequences with ≥97% similarities were classified to the same operational taxonomic units (OTUs) after the analysis using Uparse software (Uparse v7.0.1001, http://drive5.com/uparse/). Representative sequences for each OTU were screened for further annotation. For each representative sequence, the GreenGene Database (http://greengenes.lbl.gov/cgi-bin/nph-index.cgi) was employed based on the RDP Classifier (Version 2.2, http://sourceforge.net/projects/rdp-classifier/) algorithm to annotate taxonomic information. OTU abundance information was normalized using a standard of sequence number corresponding to the sample with the least sequences. For triplicate settings in the current study, mean values with the standard error were calculated using PASW statistics 18 and the figures were drawn in OriginPro 2017. Structural equation modelling (SEM) was performed with the Amos 24 software (IBM Corporation, NY, USA).

## Supplementary information

Supplementary information
