# Supplementary material for: The acetotrophic pathway dominates methane production in Zoige alpine wetland coexisting with hydrogenotrophic pathway
Source: Sci Rep. 2019 Jun 24;9:9141. doi: 10.1038/s41598-019-45590-5 (PMC6591398; doi:10.1038/s41598-019-45590-5)
Supplement: Supplementary file 1 — Supplementary information [file 41598_2019_45590_MOESM1_ESM.pdf]

The acetotrophic pathway dominates methane production in Zoige alpine wetland coexisting with hydrogenotrophic pathway

Yanfen Zhang[a, b], Anzhou Ma[a,b,*], Guoqiang Zhuang[a,b,*], Xuliang Zhuang[a,b]

[a] Key Laboratory of Environmental Biotechnology, Research Center for Eco-Environmental Sciences, Chinese Academy of Sciences, Beijing 100085, China

[b] University of Chinese Academy of Sciences, Beijing 100049, China

[*] Corresponding author: azma@rcees.ac.cn, gqzhuang@rcees.ac.cn +86 010 6292 3562

Fig. S1 The production of carbon dioxide in the methane production process. Carbon dioxide concentration in the Unamended and BES group (a), the correlation analysis between methane and carbon dioxide in the Unamended group (b) and the carbon dioxide concentration with additional methane added (c). The values are shown as the mean ± s.e.m., n = 3.

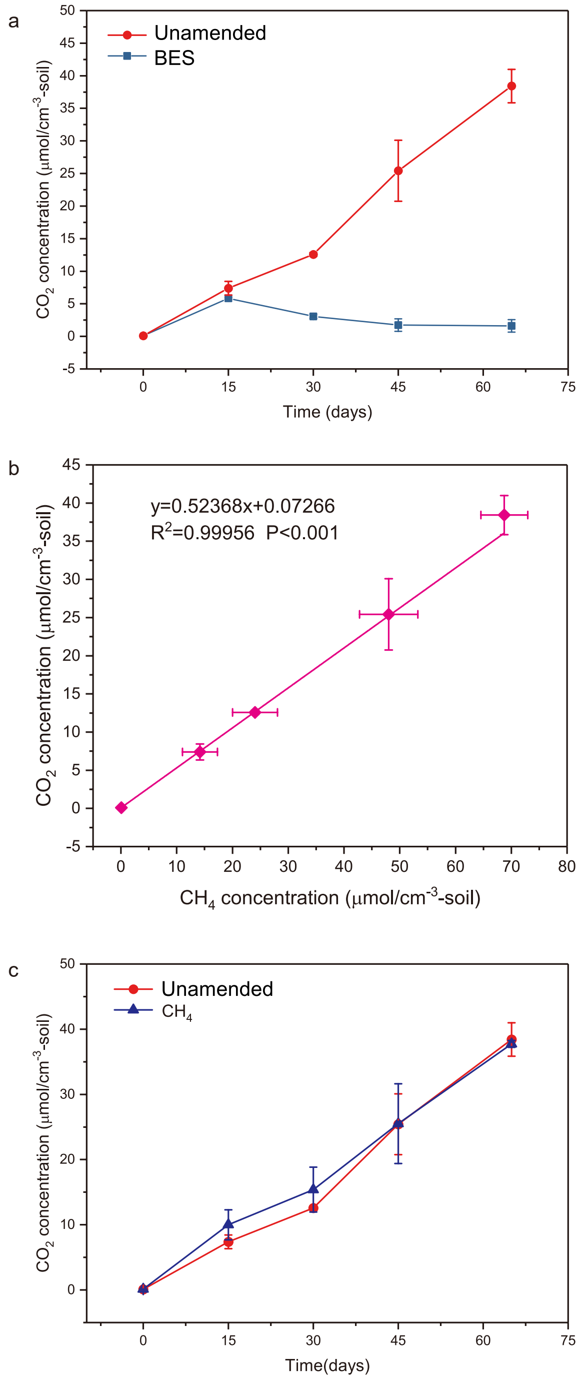

Table S1 Coordinates, pH and temperature of the sampling sites.

	U1	U2	U3	U4	U5
N	33°55′00.22″	33°54′59.85″	33°54'59.58"	33°55'00.32"	33°55'00.05"
E	102°49′18.62″	102°49′18.51″	102°49'18.95"	102°49'19.11"	102°49'18.78"
pH	7.92	7.13	8.41	7.52	7.51
T	12.6℃	13℃	13.4℃	13.5℃	13.5℃
